# Songling Xuemaikang capsules for patients with low-to-medium risk hypertension: study protocol for a randomized controlled trial

**DOI:** 10.1186/s13063-019-3308-5

**Published:** 2019-04-15

**Authors:** Yuerong Jiang, Haiyan Guo, Yue Liu, Xin Wang, Jiaqi Liang, Ruixi Xi, Ruina Bai, Dazhuo Shi

**Affiliations:** 1 Cardiovascular Disease Center, Xiyuan Hospital of China Academy of Chinese Medical Sciences, Beijing, 100091 China; 2 Graduate School of China Academy of Chinese Medical Sciences, 16 Nanxiaojie, Dongzhimen Nei, Beijing, 100700 China

**Keywords:** Hypertension, Low-to-medium risk, Songling Xuemaikang capsule, Randomized controlled trial

## Abstract

**Background:**

Hypertension is a major risk factor for cardio-cerebrovascular disease. Songling Xuemaikang capsules (SXC), a formulation of Chinese herbal patent medicine, has been used as a complementary medicine with conventional western medicine to treat patients with hypertension since 1994 in mainland China. However, the efficacy of treatment with SXC alone against hypertension remains unclear.

**Methods/design:**

This is a multicenter, placebo-controlled, double-blinded, randomized controlled clinical trial. A total of 570 patients with low-to-medium risk hypertension are randomized in a 1:1 ratio to receive SXC or placebo three times daily for eight weeks. The primary outcomes are 24-h average systolic blood pressure and average diastolic blood pressure. The secondary outcomes are daytime average blood pressure, night-time average blood pressure, fluctuation of blood pressure, hypertension control rate, traditional Chinese medicine (TCM) syndrome scores, and quality-of-life scores.

**Discussion:**

This is the first multicenter trial conducted to evaluate the efficacy and safety of TCM in patients with low-to-medium risk hypertension. Our study will provide evidence-based results of a complementary preventive measure for hypertension.

**Trial registration:**

Chinese Clinical Trial Registry, ChiCTR-IPR-17011383. Registered on 12 May 2017.

**Electronic supplementary material:**

The online version of this article (10.1186/s13063-019-3308-5) contains supplementary material, which is available to authorized users.

## Background

Hypertension is the leading risk factor for cardio-cerebrovascular disease and premature death in China [1]. Recent national surveys have reported that > 30% of the general adult population in the United States, Korea, and China has prehypertension, among which 90% have at least one risk factor for cardiovascular diseases, including coronary heart disease, congestive heart failure, ischemic and hemorrhagic stroke, renal failure, and peripheral arterial disease, above the optimal levels. Due to population aging, the incidence of hypertension is projected to increase by approximately 60% by 2025 [2–6]. The Prospective Urban Rural Epidemiology (PURE)–China subcohort study reported that the prevalence of hypertension was 41.9% among 45,108 study participants aged 35–70 years. The levels of awareness, treatment, and control were 41.6%, 34.4%, and 8.2%, respectively, among patients with hypertension [7].

Conventional antihypertensive agents are usually associated with many adverse effects. Chinese patent medicines, which are important components of traditional Chinese medicine (TCM), have been increasingly accepted by patients with hypertension. Songling Xuemaikang capsules (SXC) are a formulation for oral intake produced by Chengdu Kanghong Pharmaceutical Co. Ltd., with official approval from the China Food and Drug Administration (ratification document number: Sinopharm Z10960023). It comprises fresh pine needles, *Pueraria lobata*, and pearl powder. Studies have shown that puerarin, the main active ingredient of *P. lobata*, can reduce renin-angiotensin levels, dilate coronary and cerebral arteries, improve microcirculation, reduce vascular remodeling and ventricular hypertrophy, and exhibit anti-platelet aggregation, resulting in the promotion of endothelial cell function and the regulation of vasoactive factors [8–11]. Active calcium and various active amino acids are the active ingredients in pearl powder, which show favorable effects on the immune system, anti-oxidant effects against free radicals, and central nerve inhibition. Pine needle extract exerts anti-oxidant effects, protects the vascular endothelium, and improves blood viscosity [12, 13]. There is an urgent need for well-designed long-term studies to address the benefits of SXC for treating primary hypertension [14]. Several small-sample randomized controlled trials have shown that SXC combined with western medicine exerts superior effects over western medicine treatment alone [15, 16]; however, there is still a lack of high-quality evidence from large, blinded, randomized, placebo-controlled clinical trials. Therefore, this study is designed to evaluate the efficacy of SXC in the treatment of patients with low-to-medium risk hypertension in a multicenter, double-blind, randomized, placebo-controlled, parallel-group, superiority trial, which will provide practical evidence for the prevention and treatment of hypertension.

## Methods/design

### Study objectives

This study aims to determine the efficacy of SXC in patients with low-to-medium risk hypertension.

### Study design

This multicenter, double-blind, randomized, placebo-controlled parallel-group, superiority trial is registered in the Chinese Clinical Trials Registry (ChiCTR-IPR-17011383). It complies with the principles of the Declaration of Helsinki and Good Clinical Practice guidelines. Written informed consent will be obtained from all patients before their participation in this study; the recruited patients will be randomized to either the SXC group or the placebo group. We will rigorously follow the Consolidated Standards of Reporting Trials (CONSORT) recommendations when reporting the results [17].

The trial will be conducted in seven centers in China. A total of 570 participants will be recruited. After we acquire their consent, the participants will be enrolled in the trial, which consists of a two-week run-in period and an eight-week treatment period.

**Fig. 1:**
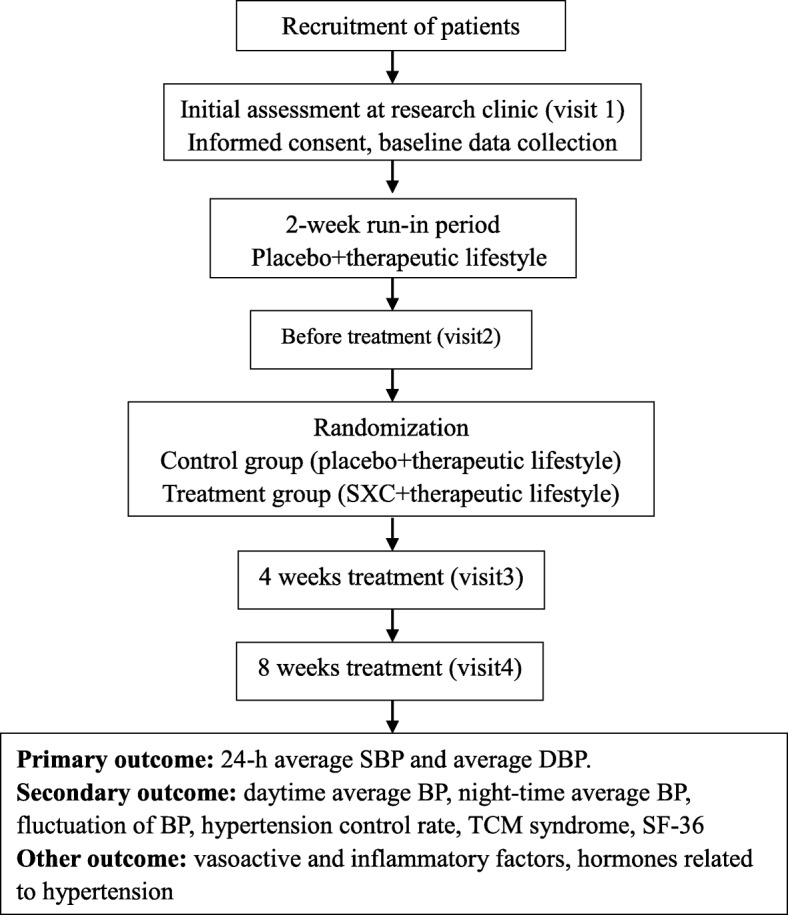
Study flowchart. SBP systolic blood pressure, DBP diastolic pressure, TCM traditional Chinese medicine, SF36 36-item Short Form Health Survey score

A completed SPIRIT checklist is available as a supplement (Additional file 1). An outline of the study procedures is illustrated in Fig. 1.

### Participants

Inpatients and outpatients in the participating centers will be screened for the selection criteria.

### Inclusion criteria

1. Primary hypertension diagnosed according to established guidelines [18]: all participants must have low-to-medium risk, grade 1 hypertension.
2. Patients must not have taken any antihypertensive drugs at all or have taken such drugs irregularly.
3. Patients must be aged 20–75 years.
4. Patients will be informed about the trial and should voluntarily sign the consent form.

### Exclusion criteria

1. Serious liver disease or alanine transaminase/aspartate transaminase values higher than the upper reference limit value.
2. Severe mental illness, serious hematopoietic disease malignancy, or a life expectancy of < 3 years.
3. Pregnant or lactating women.
4. Participation in other clinical trials within the past three months.
5. Probable or definite allergies to the ingredients of the test drug.
6. Multiple drug allergies.

### Recruitment

Patients with low-to-medium risk hypertension in each center will be screened. Each potentially eligible patient will be assessed by an attending physician to determine whether the patient should be recruited. The aim and procedures of the study and the possible side effects of the study capsules will be explained in detail to the patients. All patients will be asked to sign a written informed consent form before randomization. Neither financial nor non-financial incentives will be provided to the attending physicians or patients for enrolment.

### Randomization and treatment assignment

Patients are randomized in a 1:1 ratio through a centrally controlled, computer-generated, site-stratified, block randomization schedule. The study capsules are labeled with serial numbers; each patient will be assigned the lowest number available at each participating center. All patients, care providers, attending physicians, laboratory staff, and biostatisticians are blinded to treatment assignment as long as the database is locked. Randomization started on May 2017. The enrolment of 570 patients is expected to be completed in August 2019.

### Interventions

Eligible patients will be allocated to receive placebo for two weeks in addition to therapeutic lifestyle, including reducing sodium intake, weight control, quitting smoking, limiting alcohol intake, and physical training, according to the guidelines [18]. After the two-week run-in period, patients diagnosed with hypertension will be randomized into control or treatment groups and will be allocated to receive placebo or SXC for eight successive weeks in addition to therapeutic lifestyle changes, respectively. SXC and placebo capsules were produced and packed in a single batch by Kanghong Pharmaceutical Co. Ltd., Chengdu, China, which declares no conflict of interest relevant to this study. The drug quality is consistent with the Chinese Medicine Standard of the State Food and Drug Administration (SFDA). The placebo capsules have identical appearance and smell as those of the treatment capsules. Patients will be instructed to take a capsule orally three times daily for eight successive weeks. Any other Chinese herbal decoction or Chinese patent medicine for treating hypertension is prohibited during the trial.

### Outcomes and measures

**Fig. 2:**
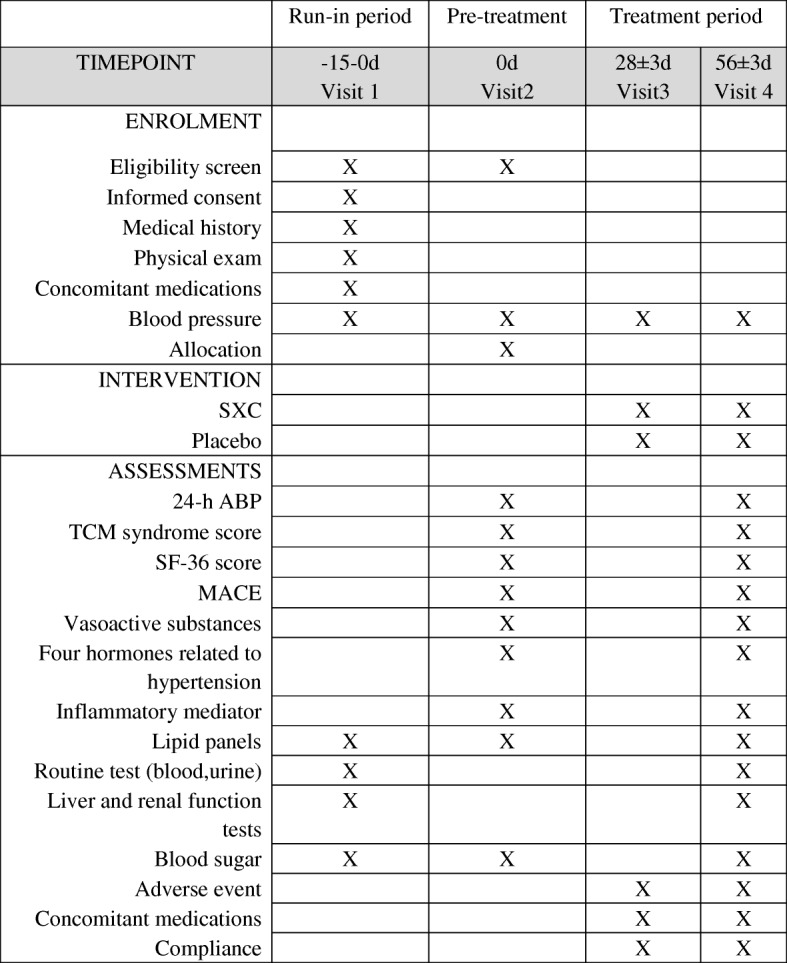
SPIRIT figure: Measurement items and points of data capture. Lipid panels represent high-density lipoprotein, low-density lipoprotein, cholesterol, and triglycerides; X represents the indicators tested in the specific time period. ABP ambulatory blood pressure, TCM traditional Chinese medicine, SF36 36-item Short Form Health Survey score, MACE major adverse cardiovascular event

The primary outcome is 24-h ambulatory blood pressure (average systolic and diastolic blood pressure). The secondary outcomes include daytime average blood pressure, nocturnal average blood pressure, fluctuation of blood pressure (24-h ambulatory blood pressure), fluctuation degree of systolic and diastolic blood pressure, control rate of hypertension (control rate of hypertension = hypertension control number/hypertension number × 100%), TCM syndrome scores, and the 36-item Short Form Health Survey score. The other outcomes include levels of vasoactive substances (nitric oxide, endothelin endothelin/nitric oxide, angiotensin converting enzyme, and homocysteine), four hormones related to hypertension (renin, angiotensin II, aldosterone, and cortisol), inflammatory mediators, and blood lipids. Safety outcomes, which include complete blood count, routine urine test, liver and renal function tests, and electrocardiograms, will also be monitored periodically. All adverse events (AEs) will be followed from randomization to the end of the trial. Items to be measured and the time window of data collection are shown in Fig. 2.

### Adverse events

AEs are defined as negative or unintended clinical manifestations following treatment. Patients will be asked to report to the investigators any abnormal reactions occurring at any time during the trial. In addition, investigators will collect information about abnormal reactions monthly. All details of related and unexpected AEs, such as time of occurrence, degree of AE, and suspected causes, will be recorded on case report forms (CRFs). Moreover, there is a data safety monitoring board to oversee the trial.

### Study-specific visits and procedures

The schedule for all study procedures for all evaluations is shown in Fig. 2. For each procedure, individuals are to be assessed by the same investigator or site personnel whenever possible. The timing of each visit is relative to randomization (day 0). Baseline measures (visit 1 and visit 2) include medical history (demographic characteristics, medical and treatment history, complicating disease, signs and symptoms, concomitant medications), clinical blood pressure, 24-h ambulatory blood pressure, vasoactive factors, four hormones related to hypertension, inflammatory mediators, TCM syndrome score, and SF-36 score. All baseline measurements will be repeated at visit 4 for all participants. AEs and outcome measurements will be recorded from visit 3 to visit 4.

### Randomization and blinding

Participants are randomized in a 1:1 ratio using a computer-generated, site-stratified, block randomization schedule. The study capsules will be labeled with sequential randomization numbers and each patient will be assigned the lowest number available at each participating center. All patients, care providers, and attending physicians will be blinded to treatment assignment until the study is completed. Duplicated blinding codes will be given to the main research institution and the manufacturer; the blinding codes cannot be revealed during the trial.

### Data entry and quality control of data

CRFs are being used for data entry and data from all participating centers will be imported into the clinical data management system (http://www.xyedc.com/). To maintain the quality of data, we will adopt valid measures to ensure information accuracy, integrity, and authenticity. First, computer logic checks will be run to identify items such as inconsistent data, missing data, and questionable values. After that, the supervisor will perform source data verification to check the consistency of the original data. Queries may be issued by the supervisor, which will be answered by the site investigators. Second, manual checks will identify more complicated and less common errors. Third, the supervisor will conduct site visits to compare the electronic database with the source documents. Identified errors will be resolved to ensure data quality. Fourth, the Data Coordination Center will be in charge of data validation.

### Sample size calculation

The sample size was calculated based on the expected reduction in hypertension. A meta-analysis has showed that SXC combined with antihypertensive drugs is more effective in lowering both the systolic blood pressure (SBP) (MD: -6.17 [− 7.86, − 4.49]; *P* < 0.01) and diastolic blood pressure (DBP) (MD: -7.24 [− 8.62, − 5.85]; P < 0.01) compared with that by antihypertensive drugs alone [14]. Furthermore, a small sample clinical trial has shown that SXC is effective in lowering the 24-h SBP by approximately 14 mmHg (mean SBP of 136.3–122.3 mmHg) after a four-week treatment period; however, the quality of the trial was relatively low [19]. According to these reports [14, 15, 19], it is assumed that the 24-h average SBP can be decreased by approximately 12 mmHg in the SXC test group and 5 mmHg in the placebo group. Assuming that the bound of value = 5 and standard deviation (SD) = 7 for the test and placebo groups, combined variance σ^2^ = 49, type I error rate (α) = 0.05, and a power of 90% (type II error rate (β) = 0.1), the sample size for one arm needed to be 258, resulting in *n* = 2 × 258 = 516 (bilateral test level α = 0.05, test efficacy β = 90%). Considering a dropout rate of 10%, a total of 570 patients needed to be allocated to attain the required number of patients for the efficacy analysis.

$$ {\displaystyle \begin{array}{l}{n}_c=\frac{\left(1+\frac{1}{k}\right)\kern0.28em {\left({\mu}_{1-a}+{\mu}_{1-\beta}\right)}^2\kern0.28em {\sigma}^2}{{\left[\left({x}_T-{x}_c\right)-\Delta \right]}^2}\\ {}{n}_t={kn}_c\end{array}} $$

### Statistical analysis

The data from all participating centers will be combined for statistical analysis of primary and secondary endpoints, as well as AEs.

The analysis will be performed at the Clinical Pharmacology Research Institute, Xiyuan Hospital, China Academy of Chinese Medical Sciences. Continuous variables will be presented as the mean ± SD. The comparability of characteristics between the two study groups will be assessed using a two-sample Student’s *t-*test for continuous variables and the χ^2^ test or Wilcoxon test, when appropriate, for categorical variables. The Wilcoxon paired signed-rank test will be used for within-group comparisons.

For all analyses, *P* < 0.05 will be considered statistically significant, and all tests will be two-tailed. All analyses will be conducted using SAS software version 9.2 (SAS Institute, Cary, NC, USA).

## Discussion

In this trial, we will assess the efficacy and safety of SXC in treating patients with low-to-medium risk hypertension. The present study is designed as a multicenter, double-blind, randomized, placebo-controlled, parallel-group superiority trial that will provide high-powered evidence regarding the efficacy and safety of SXC in treating patients with low-to-medium risk hypertension.

A previous study showed that pine needle extract has pharmacological properties that modulate hippocampal excitotoxicity-derived memory impairment under severe stress conditions [20]. In salt-sensitive hypertension, puerarin improves vascular insulin action with beneficial cardiovascular effects [21]. In addition, through the regulation of the apelin/APJ system, puerarin exerts protective effects on the development of left ventricular hypertrophy by renal hypertension [22]. The large- and small-molecule fractions of high temperature-extracted pearl extract exert a conspicuous sedative effect. The high-temperature-derived extract has been found to improve the vitality and anti-hypoxia abilities of mice. Low-temperature-extracted pearl powder increases the level of superoxide dismutase and decreases the level of malondialdehyde in the blood serum [23]. Previous studies have shown that SXC has a potential blood pressure-lowering effect, leading to the improvement of plasma levels of endothelin and nitric oxide, thereby regulating the renin-angiotensin system [24, 25]. These findings and observations provide an impetus for large-sample controlled trials to investigate the efficacy of SXC in the treatment of patients with low-to-medium risk hypertension.

For patients with low-to-medium risk hypertension, the current domestic and foreign guidelines generally recommend lifestyle improvement and monitoring of blood pressure and other risk factors 1–3 months before deciding whether to carry out drug treatment. Therefore, 10 weeks of follow-up monitoring and placebo therapy will not cause irreversible damage to the individuals [19].

There are certain limitations to this study. First, because the study is being performed in Beijing, China, it is uncertain whether the relative effects of the trial drugs would be similar in other ethnic groups. Second, the follow-up period is relatively short.

Nevertheless, this trial aims to evaluate the efficacy and safety of SXC for the treatment of patients with low-to-medium risk hypertension.

## Trial status

The trial was initiated in July 2017 and is currently open for enrolment; thus far, 120 patients have completed the 10-week follow-up. However, no analysis has been conducted since the commencement of the trial. No serious AEs have occurred to date.

## Additional file

Additional file 1: SPIRIT Checklist. (DOC 124 kb)

## Abbreviations

ABP: Ambulatory blood pressure
AE: Adverse event
DBP: Diastolic blood pressure
SBP: Systolic blood pressure
SF-36: 36-item Short Form Health Survey
SXC: Songling Xuemaikang capsule
TCM: Traditional Chinese medicine

## References

[1] He J (2016). Hypertension in China: a large and increasing public health challenge. J Hypertens.

[2] Wang Y, Wang QJ (2004). The prevalence of prehypertension and hypertension among US adults according to the new joint national committee guidelines: new challenges of the old problem. Arch Intern Med.

[3] Choi KM, Park HS, Han JH, Lee JS, Lee J, Ryu OH (2006). Prevalence of prehypertension and hypertension in a Korean population: Korean National Health and Nutrition Survey 2001. J Hypertens.

[4] InterASIA Collaborative Group (2003). Cardiovascular risk factor levels in urban and rural Thailand-The International Collaborative Study of Cardiovascular Disease in Asia (InterASIA). Eur J Cardiovasc Prev Rehabil.

[5] Wolf-Maier K, Cooper RS, Kramer H, Banegas JR, Giampaoli S, Joffres MR (2004). Hypertension treatment and control in five European countries, Canada, and United States. Hypertension.

[6] Turner AJ, Hooper NM (2002). The angiotensin-converting enzyme gene family: genomics and pharmacology. Trends Pharmacol Sci.

[7] Li W, Gu H, Teo KK, Bo J, Wang Y, Yang J (2016). Hypertension prevalence, awareness, treatment, and control in 115 rural and urban communities involving 47 000 people from China. J Hypertens.

[8] Guo YD, Xing ZL, Shu BR, Liu CL, Jia ZL (2016). Effects of puerarin combined with telmisartan on adipocytokines, activity of renin angiotensin system and insulin resistance in patients with obesity-related hypertension. Hebei Med J.

[9] Ni XQ, Li X, Zhao LH, Liu SW, Ma J, Xu JH (2005). Effect of puerarin on cardiac cholinergic innervation ischemic area in rats after myocardial infarction. Chin J New Drugs Clin Remedies.

[10] Gao L, Ji X, Song J, Liu P, Yan F, Gong W (2009). Puerarin protects against ischemic brain injury in a rat model of transient focal ischemia. Neurol Res.

[11] Pan ZY, Bao ZS, Wu ZM, Wang XM, Zheng JZ, Shen YL (2009). The myocardial protective effects of puerarin on STZ-induced diabetic rats. Fen Zi Xi Bao Sheng Wu Xue Bao.

[12] Tiwari AK, Srinivas PV, Kumar SP, Rao JM (2001). Free radical scavenging active components from Cedrus deodara. J Agric Food Chem.

[13] Chen XY (2003). Study on antioxidant properties of pine needles of Masson Pine[in Chinese]. Hubei Agric Sci..

[14] Yang XC, Xiong XJ, Yang GY, Wang HR, Wang J (2015). Songling Xuemaikang Capsule for primary hypertension: A systematic review of randomized controlled trials. Chin J Integr Med.

[15] Dong ZY, Gao Y, Wu SX, Xu L (2013). Effects of different antihypertensive regimens combined with Song ling xue mai kang capsule on antihypertensive effect. Chin J Integr Med Cardio Cerebrovasc Dis.

[16] Lan HB, Yuan HP (2015). Clinical research of Songling Xuemai Kang combined with maleic acid enalaprilat folic acid pill in treatment of essential hypertension of kidney and liver yin deficiency. Clin J Chin Med.

[17] Campbell MK, Piaggio G, Elbourne DR, Altman DG, CONSORT Group (2012). CONSORT 2010 statement: extension to cluster randomised trials. BMJ.

[18] James PA, Oparil S, Carter BL, Cushman WC, Dennison-Himmelfarb C, Handler J (2014). 2014 evidence-based guideline for the management of high blood pressure in adults: report from the panel members appointed to the Eighth Joint National Committee (JNC 8). JAMA.

[19] Jialin C (2002). Ambulatory blood pressure monitoring for the hypotensive effect of Songling Xuemaikang Capsule. Sichuan Med J.

[20] Lee JS, Kim HG, Lee HW, Kim WY, Ahn Y, Son CG (2017). Pine needle extract prevents hippocampal memory impairment in acute restraint stress mouse model. J Ethnopharmacol.

[21] Tan C, Wang A, Liu C, Li Y, Shi Y, Zhou MS (2017). Puerarin improves vascular insulin resistance and cardiovascular remodeling in salt-sensitive hypertension. Am J Chin Med.

[22] Jin G, Yang P, Gong Y, Fan X, Tang J, Lin J (2009). Effects of puerarin on expression of apelin and its receptor of 2K1C renal hypertension rats [in Chinese]. Zhongguo Zhong Yao Za Zhi.

[23] Dai J, Duan JA, Li YB, Liu R, Peng YR (2008). Investigation of sedative bioactive components and evaluation of the biological effect of freshwater pearls produced in Suzhou [in Chinese]. Chin J Biochem Pharmaceutics.

[24] Xiao MF, Zhou D (2007). Effects of “Huoxue Qianyang Granule” on rennin-angiotensin system in plasma of spontaneous hypertensive rats. Shanghai J Tradit Chin Med.

[25] Wan LH, Xiong WB, Zhu L, Liu R, Xie F, Liu JQ (2005). The antihypertensive effect and mechanism of Songling Xuemaikang Capsule on spontaneously hypertensive rats[in Chinese]. Sichuan J Physiol Sci.

